# The emerging role of WWP1 in cancer development and progression

**DOI:** 10.1038/s41420-021-00532-x

**Published:** 2021-06-21

**Authors:** Xiaoli Hu, Jiangtao Yu, Zixia Lin, Renqian Feng, Zhi-wei Wang, Gang Chen

**Affiliations:** 1 Department of Obstetrics and Gynecology, The First Affiliated Hospital of Wenzhou Medical University, Wenzhou, China; 2 Department of Hepatobiliary Surgery, The First Affiliated Hospital, Wenzhou Medical University, Wenzhou, China; 3 Key Laboratory of Diagnosis and Treatment of Severe Hepato-Pancreatic Diseases of Zhejiang Province, The First Affiliated Hospital, Wenzhou Medical University, Wenzhou, China; 4 Department of Obstetrics and Gynecology, The Second Affiliated Hospital of Wenzhou Medical University, Wenzhou, China

**Keywords:** Oncogenes, Tumour-suppressor proteins

## Abstract

Emerging evidence demonstrates that WW domain-containing E3 ubiquitin protein ligase 1 (WWP1) participates into carcinogenesis and tumor progression. In this review article, we will describe the association between dysregulated WWP1 expression and clinical features of cancer patients. Moreover, we summarize the both oncogenic and tumor suppressive functions of WWP1 in a variety of human cancers. Furthermore, we briefly describe the downstream substrates of WWP1 and its upstream factors to regulate the expression of WWP1. Notably, targeting WWP1 by its inhibitors or natural compounds is potentially useful for treating human malignancies. Finally, we provide the perspectives regarding WWP1 in cancer development and therapies. We hope this review can stimulate the research to improve our understanding of WWP1-mediated tumorigenesis and accelerate the discovery of novel therapeutic strategies via targeting WWP1 expression in cancers.

## Facts

- WWP1 mainly targets its substrates for ubiquitination and degradation.
- Targeting WWP1 could be useful for improving therapeutic outcome of cancer patients.
- WWP1 is critically involved in oncogenesis and tumor progression.

## Open questions

- What are the key drivers as the upstream factors to govern the expression of WWP1?
- Does WWP1 have a crosstalk with other NEDD4 family members?
- How to use high-screening approaches to develop the special inhibitor of WWP1 for cancer therapy?

## Introduction

Ubiquitin proteasome system (UPS) plays a critical role in regulating protein homeostasis via targeting protein post-translational modifications (PTM) [1]. Ubiquitination is a normal cellular process that one ubiquitin or multiple ubiquitins are added to the substrates, leading to protein degradation or protein trafficking [2]. This process is performed by ubiquitin-activating enzyme (E1), ubiquitin-conjugating enzyme (E2), and ubiquitin ligase (E3) [3]. Two E1 enzymes, 38 E2 enzymes, and more than 600 E3 enzymes are reported in humans. Based on their structures, E3 ligases are mainly divided into three groups: RING-type E3s, HECT-type E3s, and RBR E3s [4–7]. HECT-type E3 ligases are classified into three families according to diverse domains of N terminus: well-characterized NEDD4 family with 9 members, HERC family with 6 members, and other E3s with 13 members [8–10]. NEDD4 family displays two–four WW domains, and HERC E3 family has RLD domains, while the other E3s have neither WW nor RLD domains [11]. The WW domains bind to PPXY (phospho-Ser-Pro and Pro-Arg) motifs of the substrates and trigger the degradation [12].

**Fig. 1:**
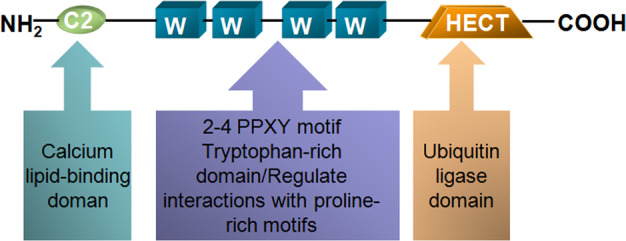
The chemical constitution of WWP1.

NEDD4 family has nine members: NEDD4-1, NEDD4-2, WWP1, WWP2, ITCH, NEDL1, NEDL2, Smurf1, and Smurf2 [12–14]. WWP1, also called TIUL1 (TGIF-interacting ubiquitin ligase 1) or AIP5 (Atropin-1-interacting protein 5), serves as a multifunction protein, which composes of an N-terminal C2 domain, followed by four WW domains and a C-terminal catalytic HECT domain (Fig. 1) [15]. Genetically, human WWP1, situated in chromosome 8q21, generates more than six isoforms resulting from alternative splicing to exert different functions [16]. Ubiquitin molecules have seven lysine residues, including K6, K11, K27, K29, K33, K48, and K63, which can be polymerized into a diverse range of linkages. K48-based chain is commonly considered as a signal label for proteasomal degradation; however, ubiquitin chains based upon other acceptor lysines, or modulation by single moiety (ies) often have a non-proteolytic effect in various important physiological processes [17]. For instance, a previous study has demonstrated that WWP1-mediated ubiquitylation of ΔNp63 was based on K63-based polyubiquitin chain, representing nonproteolytic ubiquitination. WWP1 serves as a potential modulator for the proliferation of epithelial cells [18]. WWP1 mediated PTEN polyubiquitination and repressed its dimerization and membrane recruitment [19]. Accumulating evidence indicates that WWP1 plays a critical role in regulating diverse biological processes, containing regulation of epithelial sodium channels, receptor trafficking and degradation, and viral budding [20, 21]. Moreover, the abnormal regulations of WWP1 are also involved in multiple diseases, like inflammation, neurological disorders, aging, and cancers [22]. In this review article, we will describe the association between WWP1 expression and clinical features of cancer patients. Moreover, we will discuss the various functions of WWP1 as an oncoprotein or a tumor suppressor in a variety of malignancies. Furthermore, we summarize the substrates of WWP1 and its upstream factors regulating the expression of WWP1. We also highlight the compounds as the inhibitors of WWP1 to improve the treatment outcomes of human cancers. Finally, we provide the perspective regarding WWP1 in cancer development and therapies.

## WWP1 expression is associated with poor prognosis in cancer patients

Pathologically, several lines of evidence demonstrated that WWP1 expression is aberrantly expressed in a variety of human cancers (Table 1) [22]. For example, WWP1 expression at mRNA and protein levels was reported to be commonly increased in colorectal cancer tissues [23]. High expression of WWP1 was linked to tumor size, T classification, TNM stage, distant metastasis, and poor survival [23]. One group reported that WWP1 expression was associated with single-nucleotide polymorphisms (SNPs) and copy number variants (CNVs) in osteosarcomas [24]. WWP1 mRNA levels and its copy numbers were upregulated in the oral tumor tissues [25]. High expression of WWP1 at mRNA and protein levels was also reported in gastric carcinoma tissues, which was associated with TNM stage, LNM, and invasive depth and poor prognosis in patients with gastric cancer [26]. Poor expression of WWP1 was observed in melanoma cells and melanoma tissues, which was associated with poor prognosis in melanoma patients [27]. WWP1 gene had copy number gain in 44% xenograft and cell lines that were obtained from prostate cancer. Moreover, 60% of these xenografts and cell lines had the overexpression of WWP1. Prostate tumor tissues had 31% of copy number gain of WWP1, but not frequent mutations of WWP1 [28]. Another study also revealed that WWP1 expression was increased in prostate cancer specimens compared with normal prostate specimens and PIN specimens [29]. Moreover, higher expression of WWP1 was observed in metastatic prostate cancer compared with primary prostate cancer [29, 30].

**Table 1:** Expression and prognosis values of WWP1 in human cancers.

Cancer type	Expression level of tumor	Clinicopathological features and prognosis values	Reference
Colorectal cancer	Increased	High expression of WWP1 was related with tumor size, T classification, TNM stage, distant metastasis and poor survival	[23]
Osteosarcomas	Upregulated	WWP1 expression was associated with single-nucleotide polymorphisms and copy number variants	[24, 35]
Oral cancer	Upregulated	N/A	[25]
Gastric cancer	Increased	High expression of WWP1 was associated with TNM stage, lymph node metastasis, invasive depth and poor prognosis	[26]
Melanoma	Poor expression	N/A	[27]
Prostate cancer	Upregulated	N/A	[28–30]
Breast cancer	Upregulated	Patients with only nuclear-localized WWP1 in tumors had favorable prognosis. And low/absent WWP1 level indicated the worst prognosis	[31–33]
Hepatocellular cancer	Elevated	WWP1 level was linked to tumor size, histological grade, TNM stage, vascular invasion and tumor capsule, poorer prognosis	[36, 37]
Chronic lymphocytic leukemia	Higher	High expression of WWP1 was related with adverse prognostic factors including CD38 and ZAP-70	[38]
Cutaneous squamous cell carcinoma	Augmented	High expression of WWP1 was associated with histological grade, invasion depth, lymph node metastasis and unfavorable prognosis	[39]

**Fig. 2:**
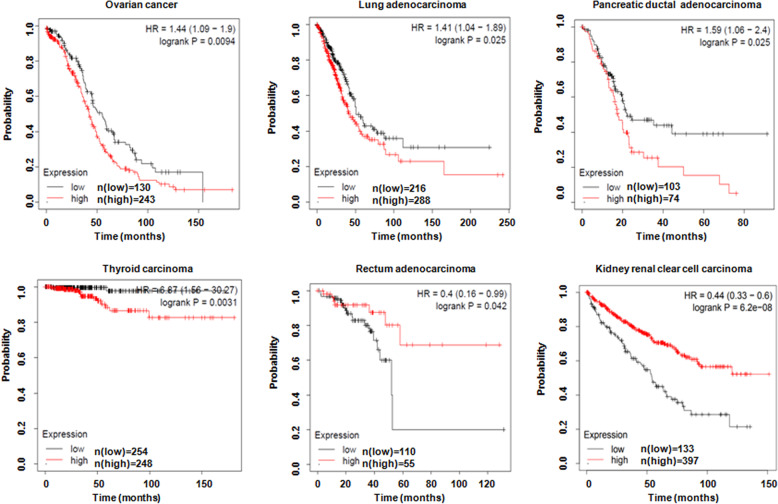
WWP1 expression is associated with overall survival in a variety of human cancers.

WWP1 mRNA was overexpressed in 58% of breast tumor cell lines, which was associated with copy number gain of WWP1 [31]. The expression of WWP1 was remarkably higher in breast tumors compared with normal tissues [31–33]. Interestingly, nuclear–cytoplasmic distribution of WWP1 might predict the prognosis of breast cancer patients. Breast cancer patients with only nuclear-localized WWP1 in tumors had favorable prognosis compared with that with both cytoplamic and nuclear WWP1 expressions [32]. Surprisingly, breast cancer patients with low or absent WWP1 expression had the worst prognosis compared with those patients with middle or high expression of WWP1 [32]. Moreover, this group reported that cytoplasmic WWP1 expression was highly expressed in breast tumor tissues and was linked to estrogen receptor alpha (ERα) and insulin-like growth factor receptor 1 (IGF-1R) expression in breast carcinoma [34]. WWP1 downregulation caused inhibition of ER levels in MCF7 and T47D breast cancer cells [34]. Similarly, WWP1 expression was higher in osteosarcoma tissues compared with matched normal bone tissues [35]. WWP1 expression at both mRNA and protein levels was elevated in hepatocellular carcinoma (HCC) specimens compared with adjacent non-tumor hepatic tissues [36, 37]. The mRNA level of WWP1 was amplified in HCC tissues [37]. Notably, aberrant high expression of WWP1 was associated with poorer prognosis in HCC patients [36]. Moreover, the expression of WWP1 was linked to tumor size, histological grade, TNM stage, vascular invasion, and tumor capsule of HCC patients, indicating that WWP1 might be an independent predicator of poor prognosis in HCC patients [36]. The high level of WWP1 mRNA expression was also found in chronic lymphocytic leukemia (CLL) patients and was positively correlated to CD38 and ZAP-70 expressions, indicating that WWP1 might be a potential marker for predicting CLL prognosis [38]. In addition, WWP1 expression was augmented in patients with cutaneous squamous cell carcinoma (CSCC) and was linked to histological grade and lymph node metastasis [39]. In line with these reports, data from Kaplan-Meier plotter (http://kmplot.com) show that dysregulation of WWP1 expression is associated with overall survival in various human cancers (Fig. 2).

## Biological functions of WWP1 in cancer

Functionally, WWP1 has been demonstrated to play either an oncogenic role or tumor suppressive functions in various types of human tumors [40]. Most studies reveal that WWP1 exerts tumor promotion functions in various types of cancers. WWP1 has been found to promote proliferation, migration and invasion, inhibit apoptosis, and enhance cell cycle in cancer cells [41].

Upregulation of WWP1 promoted proliferation and invasion of AMC-HN-8 laryngeal cancer cells [42]. Overexpression of WWP1 enhanced proliferation and migration in gastric cancer cells and facilitated tumor growth in vivo [43]. Similarly, depletion of WWP1 reduced cell proliferation in vitro and blocked tumor growth in vivo. Moreover, deficiency of WWP1 resulted in G0/G1 phase arrest and apoptosis of MKN-45 and AGS gastric cancer cells via inactivation of the PTEN/Akt pathway [26]. In line with the oncogenic role of WWP1, depletion of WWP1 by siRNA reduced growth and invasiveness of MG63 and HOS osteosarcoma cells [35]. Moreover, deficiency of WWP1 triggered G1 phase arrest and cell apoptosis in osteosarcoma cells. Mechanistically, downregulation of WWP1 regulated the expression of Bcl-2 and Bax to govern apoptosis in osteosarcoma cells. WWP1 also affected the expression of β-catenin, E-cadherin, MMP-2, and MMP-9, leading to regulating invasion of osteosarcoma cells [35]. Knockdown of WWP1 inhibited proliferation of prostate cancer cells and suppressed TFG-β-induced growth [28]. Depletion of WWP1 reduced the migration and invasion of prostate cancer cells [29]. Inactivation of WWP1 impaired MYC-driven prostate oncogenesis in the mice due to activation of PTEN [19]. Deficiency of WWP1 reduced proliferation and elevated apoptosis of oral cancer cells [25].

Similarly, knockdown of WWP1 by siRNA suppressed proliferation, colony formation, migration and invasion of HCC cells, promoted cell apoptosis, and caused cell cycle arrest at the G0/G1 phase in HCC [36]. In agreement with this report, deficiency of WWP1 repressed cell growth and stimulated apoptosis of HCC cells via upregulation of p53 and cleaved caspase 3 expression [37]. Silencing of WWP1 caused cell cycle arrest and apoptotic death of MCF7 and HCC1500 breast cancer cells via activation of caspases expression [31]. Forced upregulation of WWP1 accelerated proliferation of MCF10A and 184B5 cell lines, which are immortalized breast epithelial cells [31]. In line with this finding, another study also showed that overexpression of WWP1 in MCF10A cells promoted cell growth and colony formation, while inhibition of WWP1 repressed colony formation of T47D and MCF7 cells [32]. WWP1 knockdown in combination with tamoxifen inhibited proliferation of T47D and MCF7, and suppressed E2-mediated DNA synthesis [34]. Additionally, WWP1 promoted TRAIL resistance via inhibition of caspase-8-induced apoptosis in ERα-positive breast cancer cells [44]. Depletion of WWP1 reduced proliferation and invasion of colorectal cancer cells, while upregulation of WWP1 led to increased proliferative and invasive ability via regulation of the PTEN/Akt pathway [23]. In addition, WWP1 could mediate the resistance of doxorubicin and cisplatin in human cancer cells [45]. WWP1 expression was augmented in acute myeloid leukemia (AML) patients and inactivation of WWP1 inhibited the proliferation of AML cells and tumor growth in mice [46]. WWP1 knockdown led to cell cycle arrest and autophagy, and inhibited survival of AML cells [46]. In CSCC cells, downregulation of WWP1 impaired cell growth, blocked cell migration and invasion, induced cell cycle arrest at the G1/G1 phase and increased apoptosis in CSCC cells via suppressing phosphorylation of STAT3 and inhibiting MMP-2, cyclin D1, and Bcl-2 [39].

Interestingly, two studies exhibited that WWP1 has a tumor-suppressive function in glioma and breast cancer cells [47, 48]. WWP1 overexpression suppressed cell malignant behaviors and tumor growth in glioma xenograft mouse model [47]. WWP1 inhibited CXCL12-induced cell migration and bone metastasis in breast cancer, while knockdown of WWP1 promoted bond metastasis of breast cancer cells [48]. Without a doubt, in-depth investigations are necessary to validate the biological functions of WWP1 in cancer cells.

## Substrates of WWP1

**Fig. 3:**
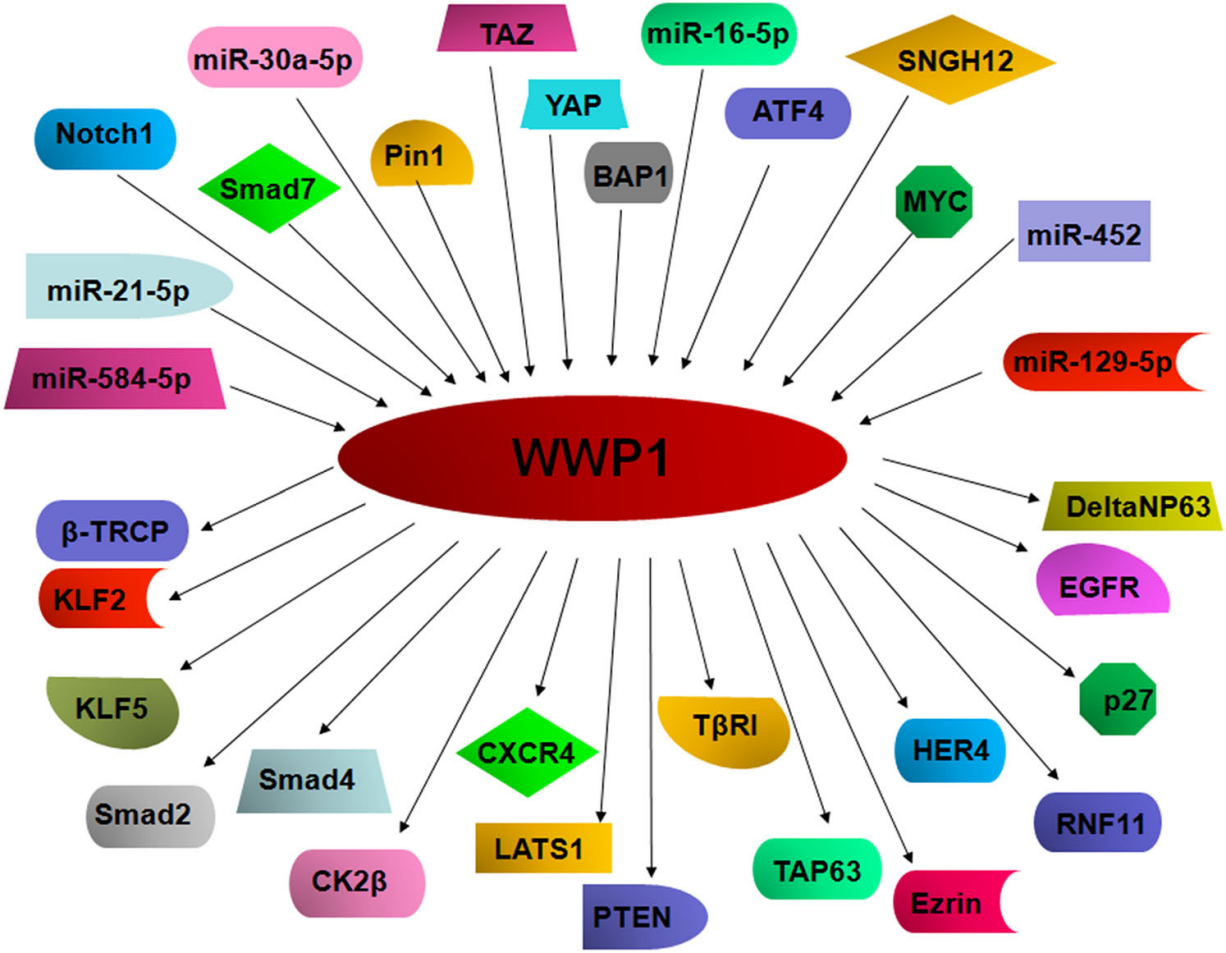
The upstream mediators and downstream substrates of WWP1.

As an ubiquitin E3 ligase, WWP1 mediates the proteasomal destruction of various substrates, including Smad2 [49, 50], KLF2 [51], KLF5 [52, 53], TGF-β (transforming growth factor-β) receptor type 1 (TβR1) [54], ErbB4/HER4 [55], p63 [45], and LATS1 [56] (Fig. 3). Here, we briefly describe the multiple substrates of WWP1 and display how the substrates are degraded by WWP1 and lead to controlling carcinogenesis.

### Krüppel-like factor 2 and 5

The transcription factor KLF2 and KLF5 critically participate into human oncogenesis. One study revealed that WWP1 can bind to the inhibitory domain of Lung Krüppel-like factor (LKLF/Krüppel-like factor 2) and inhibit its transactivation, indicating that WWP1 repressed KLF2 transactivation [51]. Chen et al. [52, 53] reported that WWP1 acted as an E3 ubiquitin ligase for the ubiquitination and degradation of KLF5. WWP1 can bind with KLF5 and its catalytic cysteine residue is required for this degradation. A PY motif in KLF5 domain is important for binding with WWP1 for its degradation [52]. Moreover, this group identified that KLF5 destruction via the proteasome might be governed in a ubiquitin-independent way [57].

### Smad2 and Smad4

WWP1 has been identified to inhibit TGF-β signaling via regulating degradation of Smad2 and activated receptor [49, 50]. WWP1 interacted with Smad7 and triggered degradation of the activated type I receptor. WWP1 also can bind with Smad2 and TGIF, leading to enhancement of Smad2 degradation. Upregulation of WWP1 attenuated TGF-β-mediated growth arrest, while depletion of WWP1 led to inhibition of Smad2 degradation and promoting TGF-β-induced gene expression [49]. In addition, WWP1 induced nuclear export of Smad7 and suppressed TGF-β-mediated Smad2 phosphorylation, leading to negative regulation of the TGF-β pathway [50]. WWP1, Smurf2, and NEDD4-2 cooperated with Smad7 to downregulate Smad4 via proteasome degradation [58].

### CK2β

One study has revealed that WWP1 and CHIP are critical E3 ligases for targeting CK2β degradation [59]. CK2 is critically involved in TGFβ-mediated EMT and promoted cancer metastasis. TGF-β increased CK2 activation and inactivation of CK2 blocked TGF-β-induced EMT [59]. Overexpression of WWP1 reduced the CK2β protein levels, while MG132 abolished WWP1-involved degradation of CK2β. WWP1 interacted with CK2β and promoted its ubiquitination and degradation [59]. TGF-β enhanced WWP1-mediated destruction of CK2β, which was abrogated by the absence of WWP1 expression [59]. Moreover, WWP1 was involved in TGF-β-mediated EMT via regulating CK2β degradation.

### CXCR4

CXCR4 is a chemokine receptor that binds the CXCL12 (also known as SDF-1), which plays a pivotal role in tumorigenesis and cancer metastasis [60]. One study showed that knockdown of WWP1 elevated the expression of CXCR4 in MDA-MB-231 breast cancer cells. CXCL12 induced CXCR4 degration in MDA-MB-231 cells, but not in WWP1-depletion cells. WWP1 knockdown increased mobility of MDA-MB-231 cells induced by CXCL12 [48]. Moreover, WWP1 controlled CXCR4 lysosomal localization in response to CXCL12 [48]. In summary, WWP1 govern CXCL12-mediated lysosomal degradation of CXCR4, leading to regulation of cell migration and bone metastasis in breast cancer.

### LATS1

The large tumor suppressor (LATS1) is a key factor in the Hippo signaling pathway, which is involved in carcinogenesis and tumor progression [61, 62]. WWP1 was validated as an E3 ligase to negatively regulate LATS1 expression. Moreover, WWP1 promoted LATS1 ubiquitination and degradation, leading to promoting proliferation of breast cancer cells [56]. Therefore, inhibition of WWP1 could be a promising approach for activation of LATS1 and further blocking growth of breast cancer cells.

### Other substrates

WWP1 was reported to induce the degradation of TβRI in conjunction with Smad7 [54]. One study demonstrated that WWP1 can bind and ubiquitinate and destroy the TAP63 and DeltaNP63, two different forms of p63 protein [45]. Another study reported that WWP1 targeted HER4 and membrane HER4, but not nuclear HER4, for degradation [55]. In support of this study, one group also observed that WWP1 suppressed the expression of ErbB4/HER4 via ubiquitination and degradation in breast cancer [63]. WWP1 also targeted p27 protein for ubiquitination and degradation, contributing to G0/G1 cell cycle arrest in AML cells [46]. WWP1 increased the expression of ErbB2 and EGFR via interacting with RING finger protein 11 (RNF11) and promoted cell growth. WWP1 ubiquitinated RNF11, but it did not cause degradation of RNF11 and cellular localization [64]. Therefore, WWP1 exerted oncogenic functions via inhibiting RNF11-triggered downregulation of ErbB2 and EGFR. Likely, WWP1 ubiquitinated Ezrin and did not cause the degradation of Ezrin, but WWP1 upregulated the expression of Met level and regulated hepatocyte growth factor receptor activity [15]. WWP1 caused PTEN polyubiquitination and blocked its dimerization and membrane recruitment, leading to activation of Akt [19]. Moreover, this group identified that WWP1 K740N and N745S alleles were enriched in colon cancer patients. These WWP1 variants are one of reasons to increase enzymatic activation of WWP1 and consequent inhibition of PTEN activity [65]. However, one study did not find the similar results and challenged the point that K740N and N745S WWP1 variants facilitated tumorigenesis via promoting PTEN ubiquitination [66]. Recently, WWP1 was discovered to bind with EGFR and increase its ubiquitination and enhance EGFR stability, resulting in enhanced lung cancer progression [67].

## Upstream factors of WWP1

Accumulating evidence has revealed that WWP1 expression level is regulated by several factors and noncoding RNAs. In the following paragraphs, we describe how multiple factors such as YAP, TAZ [68, 69], BAP1 [27], Notch-1 [70], and noncoding RNAs, including miR-16-5p [71], miR-21-5p [71], miR-30a-5p [47], miR-129-3p [43], miR-129-5p [43], miR-452 [29], miR-584-5p [72], and lncRNA SNGH12 [42], regulate the expression of WWP1 in human cancer (Fig. 3).

**Table 2:** Main cancer-related substrates of WWP1.

Substrate	Modulation	Roles	Reference
KLF2	Binding KLF2 and inhibiting its transactivation	Not discussed	[51]
KLF5	Degradation in a ubiquitin-independent way	Not discussed	[52, 53]
Smad2	Degradation	Inhibition of TGF-β signaling	[49, 50]
Smad4	Degradation	Attenuated TGF-β signaling	
CK2β	Ubiquitination and degradation	Inhibition of TGF-β-induced EMT	[59]
CXCR4	Limitation of degradation	Enhancement of cell migration and bone metastasis in breast cancer	[48]
LATS1	Ubiquitination and degradation	Promoted proliferation of breast cancer cells	[56]
TβRI	Polyubiquitination and degradation	Inhibited TGF-β cytostatic signaling, and exhibited carcinogenic properties	[54]
TAP63	Ubiquitination and degradation	Restrained apoptosis and sensitivity to doxorubicin and cisplatin in colon cancer cells	[45]
DeltaNP63	Ubiquitination and degradation	Increased doxorubicin-induced apoptosis in breast cancer cells	[45]
ErbB4	Ubiquitination and degradation	Tumor inhibition in breast cancer	[55]
p27	Ubiquitination and degradation	Promoted leukemic cell growth	[46]
RNF11	Ubiquitination, not degradation	Enhanced proliferation and survival of cancer cells	[64]
Ezrin	Ubiquitination, not degradation	Increased Met level and further promoted proliferation of cancer cells	[15]
PTEN	Polyubiquitination	Promotion of cancer development	[19]
EGFR	Ubiquitination and stabilization	Enhanced NSCLC stemness and inhibited its chemosensitivity	[67]

### YAP and TAZ

Both Yes-associated protein (YAP) and TAZ antagonized degradation of KLF5 by WWP1, resulting in enhancement of proliferation of breast cancer cells [68, 69]. YAP and TAZ, two key factors of Hippo signaling pathway, blocked the WWP1-mediated KLF5 destruction because YAP and TAZ and WWP1 can bind to the PY motif of KLF5 [68, 69]. Overexpression of TAZ increased KLF5 and its target FGF-BP expression, while downregulation of TAZ exhibited the opposite effects and retarded growth of 184A1 breast cells and HCC1937 breast cancer cells [68]. Upregulation of YAP elevated KLF5 protein levels and FGFBP1 and ITGB2 expressions, two KLF5 downstream targets, contributing to proliferation and survival of MCF10A and SW527 breast cells [69]. Thus, YAP and TAZ exert tumor promotion in part via blocking KLF5 from WWP1-involved degradation and stabilizing KLF5 activity.

### BAP1

WWP1 targets KLF5 for degradation via K48-linked ubiquitination in melanoma cells. KLF5 enhanced malignant phenotypes and suppressed autophagy of melanoma cells via activation of PI3K–AKT–mTOR pathways [27]. BAP1 blocked WWP1-induced degradation of KLF5 and led to upregulation of KLF5 and promoting melanoma development [27]. In addition, BAP1 as a deubiquitinase enhanced cell proliferation and metastasis via deubiquitinating KLF5 in breast cancer cells [73]. BAP1 interacted with KLF5 and led to its stability and exerted its oncogenic function in breast cancer. Therefore, the relationships among BAP1, WWP1 and KLF5 need to be further investigated.

### MiRNAs

One study showed that miR-16-5p was increased in the feces in inflammatory bowel disease (IBD), including ulcerative colitis and Crohn’s disease, whereas miR-21-5p was highly expressed only in ulcerative colitis patients, indicating that these two miRNAs might be biomarkers for predicting IBD [71]. WWP1 could be a potential target of miR-21-5p and miR-16-5p in IBD, and promotes the initiation and progression of IBD-related colorectal cancer [71]. One group demonstrated that WWP1 is a direct target of miR-30a-5p in glioma [47]. WWP1 mRNA expression was negatively associated with miR-30a-5p expression in glioma specimens. Moreover, NF-kappaB p65 upregulated the expression of miR-30a-5p via interaction of NF-kappaB RelA subunit and the miR-30a-5p promoter region, leading to inhibition of WWP1 and promoting glioma malignant phenotype [47]. Interestingly, WWP1 upregulation also reduced miR-30a-5p expression and inhibited p65 expression in glioma cells. Therefore, a miR-30a-5p/WWP1/p65 feedback loop was exhibited to regulate development of glioma [47].

It has been identified that miR-129-5p and miR-129-3p targeted WWP1 in gastric cancer cells [43]. Moreover, miR-129-5p can interact with WWP1 mRNA at its CDS region. Furthermore, miR-129-5p and miR-129-3p repressed cell proliferation and migratory ability via suppression of WWP1 in gastric cancer [43]. WWP1 has been reported to be a direct target of miR-452 in prostate cancer cells [29]. Specifically, miR-452 expression was decreased in prostate cancer patients, while WWP1 was highly expressed in patients with prostate cancer. WWP1 expression was negatively correlated with miR-452 expression levels [29]. Moreover, patients with low expression of miR-452 had a poor survival rate in prostate cancer. Overexpression of miR-452 suppressed migratory and invasive capability of prostate cancer cells partly via downregulation of WWP1 [29]. Targeting miR-452 might be a potential approach to regulating WWP1 for treating prostate cancer.

Overexpression of miR-584-5p suppressed proliferation of gastric cancer cells and increased apoptosis [72]. Moreover, WWP1 was identified as a direct downstream target of miR-584-5p in gastric cancer cells. Upregulation of WWP1 abolished the effects of miR-584-5p overexpression on gastric cancer cells, while depletion of WWP1 impaired the function of miR-584-5p inhibitors [72]. Consistently, WWP1 expression was negatively associated with miR-584-5p expression in gastric cancer specimens. Mechanistically, miR-584-5p decreased WWP1 expression, leading to accelerating senescence and activating TGF-β pathway in gastric cancer [72].

### LncRNA SNGH12

LncRNA SNGH12 (small nucleolar RNA host gene 12) elevated cell proliferation and invasiveness via acting as a sponger of miR-129-5p and subsequent upregulation of WWP1 in laryngeal cancer cells [42]. WWP1 was positively governed by SNHG12 at the both protein and mRNA levels. In addition, WWP1 was negatively controlled by miR-129-5p in laryngeal cancer cells [42]. This study suggested that WWP1 was regulated by SNHG12/miR-129-5p axis in laryngeal cancer.

### Other upstream factors

Estrogen promoted the interactions between ERβ, WWP1 and KLF5, leading to promotion of KLF5 degradation in prostate cancer cells [74]. Activating transcription factor 4 (ATF4) suppressed the expression of WWP1 mRNA under oxidative stress, leading to the stability of LATS1 and inactivation of YAP and promotion of cell death [75]. WWP1 autoinhibition was relieved via interacting with Smad7, leading to promoting TβRI degradation [54]. Upregulation of Smad7 inhibited the abundance of WWP1, whereas knockdown of Smad7 led to an increase of endogenous WWP1 [54]. Moreover, Smad7 expression triggered WWP1 polyubiquitination and degradation via blocking the interaction between C2 or WW and HECT domains [54]. Peptidyl-prolyl isomerase Pin1 interacted with p63α and impaired the binding between p63α and WWP1, resulting in inhibition of WWP1-mediated p63α degradation and promotion of cell proliferation and tumor formation [76]. WWP1 interacted with the cytoplasmic domain of Notch1 and Notch1 regulated the nuclear localization of WWP1 [70]. Moreover, the MYC gene is one of the most commonly deregulated oncogenic genes in the formation, development, and progression of human carcinomas [77]. Recently, Lee et al. [19] discovered that MYC gene significantly increased the expression level of WWP1, and WWP1 depletion markedly reactivated PTEN function in prostate cancer, resulting in the suppression of the PI3K–AKT signal pathway and MYC-mediated carcinogenesis.

## Targeting WWP1 for cancer therapy

Bortezomib, a proteasome inhibitor, prevented development and bone metastasis via suppression of WWP1, Smurf1, and Smurf2 in prostate cancer [30]. Bortezomib inhibited the mRNA and protein levels of WWP1 in prostate cancer cells, leading to cell growth suppression [30]. Indole-3-carbinol (I3C) was reported to inhibit WWP1 via binding with the WWP1 HECT domain, leading to PTEN plasma membrane accumulation, suggesting that I3C might be a potent inhibitor of WWP1 [19]. DNA damage chemotherapeutic compounds increased the mRNA and protein levels of WWP1 [45]. We believe that more specific inhibitors of WWP1 will be discovered for targeted therapy of human cancer.

## Conclusions and perspectives

In conclusion, a line of evidence has highlighted the significance of WWP1 in tumorigenesis mainly via regulating numerous substrate turnovers (Table 2). It is important to mention that WWP1 displays dual roles to promote or inhibit cancer initiation and progression. Although studies show the functions of WWP1 and underlying mechanisms, some crucial questions need to be answered to fully elucidate the molecular insight into WWP1-involved carcinogenesis. For instance, most studies discovered the substrates of WWP1 in cancer cells. What are the key drivers as the upstream factors to govern the expression of WWP1? Does WWP1 have a crosstalk with other NEDD4 family members? It is better to use WWP1 engineered mouse models to define the functions of WWP1 in oncogenesis. The special inhibitors of WWP1 are not available so far. How to use high-screening approaches to develop the special inhibitor of WWP1 for cancer therapy? Downregulation of WWP1 elevated the expression of DeltaNP63a in the MCF10A and 184B5 breast epithelial cells and caused resistance to doxorubicin-mediated apoptosis, but also upregulated TAP63a levels and caused apoptosis, and reduced resistance to doxorubicin and cisplatin in HCT116 colon cancer cells [45]. This study clearly suggested that WWP1 plays a different role in a context-dependent manner via targeting two types of p63 proteins for destruction [45]. Similarly, membrane HER4 was degraded by WWP1, while nuclear HER4 was destructed by the anaphase-promoting complex, indicating that WWP1 might target its substrates in specific cellular compartments [55]. Therefore, it is pivotal to design and develop medicines targeting WWP1 in special tissues of cancer patients. These investigations will remarkably improve our understanding of WWP1-mediated tumorigenesis and promote the discovery of novel therapeutic strategies via regulation of WWP1 expression in cancers.
